# Oral Foci of Infection from a Dentist’s Standpoint*Read before the Cincinnati Dental Society, May 17, 1919, and reprinted from the “Dental Summary” for July.

**Published:** 1919-09

**Authors:** H. H. Schuhmann

**Affiliations:** Chicago, Ill.


### ORAL FOCI OF INFECTION FROM A DENTIST'S STANDPOINT*

BY H. H. SCHUHMANN, M.D., D.D.S., CHICAGO, ILL.

The question of oral foci of infection is by no means a new one. It has been thoroughly discussed by many writers until it has become almost trite. Nevertheless, in spite of all this discussion, the wide difference of opinion that exists upon this subject is simply appalling. Why is it that no definite basis of agreement has been arrived at between

* Read before the Cincinnati Dental Society, May 17, 1919, and reprinted from the "Dental Summary" for July.

the medical and dental diagnostician as to the real significance of oral foci of infection? Even at this late date some are inclined to attach very little importance to it, while others go to the opposite extreme and place the greatest significance upon it, believing that foci of infection about the roots of the teeth are the sole source of disease, to the practical exclusion of all other foci.

Such wide difference of opinion does not exist among any other branches of medicine. What is there that hinders us from obtaining some mutual understanding on this subject? The answer to this question lies in the fact that for years there has been something radically wrong in the instruction received in pathology, and the great lack of instruction in bacteriology in our colleges. As long as we possess limited information and entertain narrow points of view, we can not hope to arrive at a scientific conclusion on this important subject. The number of physicians and dentists who are satisfied in making an empirical diagnosis gradually is declining, for they have learned that such a diagnosis generally is wrong, with the result that the treatment is usually disappointing in its result.

Not many years ago we gave very little thought to the etiology of, for example, pyorrhea alveolaris. Today we assign at least four causes to this condition, three of which etiological factors are well known to all of you: chronic irritation due to tartar, decay and defective dental work, unsanitary conditions about the mouth, with resultant lodgment and putrefaction of food particles, constitutional and metabolic reactions, bacterial and parasitic causes. This latter factor the writer particularly desires to emphasize and develop. The organisms generally found in this condition are the various members of the streptococcic group: spirochaeta, and also fusiform bacilli, often found in typical cases of Vincent's angina, many types of putrefactive bacilli, amoeba, etc. The micro-organisms present in septic pockets are very numerous. Upon examining the superficial pus in pyorrhea pockets, we find a number of the above-named organisms present. By wiping away the superficial discharge and making a direct smear or a culture of the deeper parts, we find the streptococci and staphylococci predominating, the streptococci often being present in pure culture, either as the streptococcus viridans or the streptococcus hemolyticus type.

While it is very unlikely that any of these organisms are the specific causative agents, it is agreed generally that most of them are pathogenic, and under suitable conditions are capable of producing acute and chronic inflammation. It is of great importance always to trace focal infection and not merely to make a snap empirical diagnosis, ascribing it to some dental trouble, just because such a point of view happens to be the prevailing and fashionable viewpoint at the time. It is needless to state the utter worthlessness of any such diagnosis, made without all the substantiating evidence obtainable from laboratory findings. One must remember constantly that it is unlikely that any of the organisms mentioned ever cause local or systemic infections, unless at the same time there is some *existing abnormal condition* present, which *diminishes the resistance* to the invading organism.

In all questions involving infections it is necessary to take into consideration the fact that while various organisms, according to Vaughan, of Ann Arbor, have their individual toxins which may be more or less harmful, they all have one *common* toxin, namely, their albumose constituents, the latter being largely contained even in organisms of a saprophytic nature; and, *secondly,* we must consider the entire potentialities of the body, for they all come into play. It is therefore fallacious to confine one's investigations to a limited sphere, without giving due consideration to the entire body forces. In studying these cases it is absolutely necessary to avail oneself of all scientific knowledge and laboratory research. It is just as wrong to belong to the ultra-conservative group, who look upon this as a passing fad, as it is to belong to the overzealous class, who run every idea into the ground, simply because the opinions are based upon insufficient experimental facts. The class to which most of us belong, I am glad to state, has an open mind always on the alert for new and well-substantiated facts, and are continually guarding against unwarranted generalizations and conclusions.

We may define a focal infection as a circumscribed lesion of a bacterial nature, resulting in infection of a contiguous or noncontiguous part by the same bacteria of their toxins. From this definition it is clear that even if the presence of bacteria in a circumscribed area is demonstrated, *that of itself does by no means constitute a focus of infection.* We are all familiar with the long-established fact that bacteria may be carried about for long periods of time without creating bodily infection, and without even producing any local symptoms. For example, the organisms of diphtheria, pneumonia, spinal meningitis and many others are frequently harbored by perfectly healthy people for great lengths of time, and who are entirely ignorant of the fact that they carry organisms of such a highly-infectious nature. Whether this peculiar condition is due to the fact that the organisms present happen to be in such a state that they are harmless, or are walled off, or the host does not seem to be in a receptive state for their ill effects, matters not. One thing we must admit, namely, that *these organisms although present in the patients do not necessarily cause local or systemic disturbances and do not constitute foci of infection.* Failure to recognize these facts will inevitably lead to much misdirected effort and to disappointing treatment.

It is important to note that in growing the organisms which interest us in the laboratory, various kinds of media must be used; but for the development of streptococci only blood agar media is suitable; otherwise, the streptococcus viridans, for example, will not grow at all. In the cases that have come under the writer's observation he has obtained excellent results by using 10 c. c. of ox blood in 90 c. c. of 0.8 per cent. of agar. On this media the streptococcus viridans will appear usually after twenty-four hours' growth at body temperature, showing the characteristic small green colony.

It might be worth while to say a few words about the spirillum, which is associated with a fusiform bacillus found in large numbers in Vincent's angina. These organisms are found in normal individuals in the gingival margins around the teeth, and within the tonsils. They are sometimes found in almost pure culture in several cases of pyorrhea. Vincent's angina may progress rapidly, causing destruction of the soft tissues and alveolar process, even in patients whose teeth and gums before such infection were fairly normal. It is more often seen, however, in individuals who are careless in the care of their teeth. The condition yields readily to local treatment. The organism, together with the spirillum, can be best stained for microscopic examination by using carbol fuchsine for one minute, heating the slide gently after adding the stain.

Should an oral focus of infection actually be demonstrated, then there are two other factors that come into play before the host will suffer from constitutional derangements of any kind, namely, the bacteria or their toxins must enter the blood stream, and the local area to become infected secondarily, *must be in such a state of lowered resistive powers* that it will receive the organisms or their toxins and permit of the further propagation of the bacteria. In other words there must be a *locus minoris resistentia.* The presence of bacteria somewhere in the oral structure, with a synchronous appearance of arthritis or endocarditis, is *by no means any evidence that one is due to the other.* It is true that under proper auspices an alveolar infection might eventually produce endocarditis; but let us not overlook the fact that while alveolar infections frequently are present in the young, particularly in the children of poor districts; it nevertheless is but *very rare*

that we find these children suffering from endocarditis. It would seem, therefore, definitely to associate endocarditis, or for that matter arthritis, *always* with *dental* foci of infection is at least very debatable.

It has been claimed that in some cases of skin diseases as, for example, eczema, oral infection has played a distinct part. Various authors have reported cases of disseminated sclerosis and iritis as probably due to this cause, without, however, giving any substantiating evidence.

About a year ago it was reported that rabbits had been injected with streptococcus viridans obtained from alveolar infections, and that these organisms had in *all* cases, and by virtue of their *peculiar selective characteristics,* attacked the serous and synovial membranes of these animals, *all* of them showing arthritis and endocarditis. The attempt was made thereby to prove that the streptococci contained in granulomas at the root ends of teeth had a very definite bearing on sequential results; namely, endocarditis and arthritis. These experiments have been widely discussed by men who lack scientific training, and who accepted these statements as facts. These experiments were made to prove the *selectivity* of organisms; in other words, when organisms were obtained from such locations as granulomas and abscesses at the apices of teeth, their introduction into the ear vein of rabbits being invariably followed by attacks by these organisms on their *synovial* membranes, shows their tissue selectivity for this particular tissue. Such findings, however, *do not prove any such fact.* It has since been found that *any* organism introduced in such manner attacks the synovial membranes, producing arthritis and endocarditis and many other symptoms of sepsis.

Evidently it is not the selective action of organisms which produces these results, but it is the fact that these particular tissues are very amenable to such attacks by *any* organisms; *not* a chemotactic process between the organism and the tissue at all. Many investigators have proven repeatedly the fallacy of the selective factor, and have demonstrated the same end results, namely, arthritis and endocarditis in rabbits, by injections of bland chemicals or, as has been shown by Professor Rufus, of New York, even by the introduction of plain egg albumen. Furthermore, we all know that rabbits are particularly susceptible to the activities of streptococci, just as other animals show particular susceptibility to other organisms as, for instance, the white mouse to the pneumococcus.

Rheumatism has for a long time been ascribed by the medical profession at different periods to different organisms, and at present the etiologic factor seems to be commonly accepted to be the streptococcus viridans. The name "rheumatism" simply describes a complex of symptoms, such as arthritic disturbances, changes of temperature and involvement of all serous membranes, including that of the endocardium. The more definite information which we are obtaining from bacteriologic laboratories tends to show that some different forms of arthritis are due to specific organisms; for example, such as is found in gonorrheal arthritis, in typhoid and diphtheritic arthritis. These are classes of arthritis which certainly do not depend for their etiology on streptococcus infections, derived from oral foci, and as time progresses no doubt various other forms of arthritis will in their name be linked to proper specific organisms. It is, therefore, clear that all cases of arthritis are by no means due to the streptococcus viridans and *certainly not to those only found in oral infection.*

An infection, as we have previously stated, is a morbid bacterial process in which the organisms or their toxins have found their way into the blood stream and by a process of dissemination have established similar bacterial seats of infection in tissues, either contiguous or noncontiguous. It would seem, however, that when we speak of infections we usually refer to the absorption of bacteria and only seldom refer to what is at least as important, namely, toxemia, due to the general toxin contained by all organisms. Evidently the dividing line between endoand exo-toxins can not be drawn too definitely. Toxemia refers to the absorption of bacterial poison. It is not necessary, therefore, for a secondary infection to take place, that bacteria must be transferred from one situation to another, but the harmful results of same may be due to their general toxin. This is equally true of saprophytic organisms. Organisms may do their harm directly to the tissues, or their toxins may so *hinder the defensive powers of the body* as to permit or invite the future attacks of organisms from an entirely different source.

The whole surrounding media of the hormones of the internal glands may thus be changed by the results of toxic infection; and as our anti-body production is in a large measure dependent upon the proper activity of these hormones, it may be much decreased by such effects. *These toxins do not necessarily have to come from oral foci of infection, as there are many other sites of possible foci of infection which may produce just such toxins.* This may account for some of the obscure cases of arthritis which are produced by toxins lowering the resistive power of the serous and synovial membranes, so that bacterial invasion from the same or from still another source is invited.

Naturally, deep-seated infections and general sepsis must have their cause in some localized infection somewhere in the body. The same is true of general toxemia. We might mention aside from the dental regions, however, many others, such as otitis media, pyelitis, tubercular processes, intestinal disturbances, all of which may be due to some other local process or to a toxemia indirectly responsible and due to some other local infection.

There are so many angles to this question that it seems astonishing that so much stress should have been laid upon dental infections in particular, when in reality if we consider these in particular they should, according to all scientific reasoning, be even less apt to be the etiologic seat of such secondary infections for which they usually are blamed. Particularly in small granulomas at the end of a tooth root we find bacteria contained in a very small, thick, fibrous sac, surrounded for months and sometimes for years by their own offal, with very little fresh nutrient material, almost entirely walled off. We know nothing about the fluid content as regards alkalinity, or glycogen content, data which are of the greatest importance if the virility of the organism is to be considered. We know nothing of the virility of the organisms as to their power to traverse the thick envelopes of fibrous tissue. If it is a fact that these organisms are really pathogenic, due to their specific toxin, and are constantly and slowly piercing the fibrous wall and gaining access to the blood stream, would we not, by such frequent gradual infiltrations, be justified in the belief that such an osmosis would tend to produce a high degree of tolerance, inasmuch as this gradual invasion of bacteria would act much like the very best sort of vaccine? As you see, there are a great many vital questions to be considered, and to follow blindly new theories without investigations should not be the road for the scientifically-developed mind to travel. There are many other avenues of secondary infection which rarely are thought of, such as the results of infection from eruptive fevers. If it is definitely determined that the source of a morbid state lies in a certain focus of infection let it be our duty, and that of the physician, to locate that source and not ascribe it to a certain locality, merely because the prevailing idea happens to ascribe most diseases to a bacterial infection from a certain localized area.

In the young, tonsillar and middle-ear infections surely play a great role in the general status of health, and there again the mere presence of organisms is in nowise proof that these situations are the sites of focal infection. We find in children frequently, and in adults also, for that matter, at times a large increase in colon bacilli. Does that mean a focus of infection? No; it is even desirable that these colon bacilli be present in their proper location. We know that middle-ear infections may be present for a long time without causing the slightest local or constitutional trouble. Thus, streptococci and diphtheria bacilli, as well as a large number of other pathogenic organisms, may be present for weeks and even years without causing any ill effects.

It would seem to the writer that in order to arrive at some sane avenue of approach to scientifically investigate the matter of oral infection and its consequences, we are obliged to view the matter from two widely different avenues of bacterial location. Namely, the *one* a filth condition about the teeth caused by unhygienic neglect, or a result of pyorrheal infection; in other words, an *open infectious area* from which the bacterial infection easily can spread to adjacent tissues, such as the tonsils, nasopharynx and alimentary tract, and readily can cause invasion of bacteria with consequential albumose poisoning. And the *other*, that condition found at times at the root ends of teeth where bacteria are supposed to be in granulomatous tissue about the apices of devitalized teeth; and this again, in the writer's opinion, should be definitely subdivided into two classes, namely, *one* showing a very slight area of radio translucency, and the *other* a large area of that sort which to the writer's mind would indicate a definitely-spreading, invasive infection of a pathogenic nature; and in this category the writer would include such spreading infections which at times are seen at the apices of devitalized teeth, reaching into the antrum or into the inferior dental canal, these later unquestionably being dangerous seats of infection.

As far as the writer's clinical observation goes, he is forced to admit that the greatest number of those spontaneous cures with which he has become acquainted, were achieved by the prophylactic treatment or extraction of such teeth as are enumerated in the first category, where secondary infection resulting from the local filth condition has followed, and where the infection has occurred on the basis of a toxemia, due to albumose, but he has found that in such cases where devitalized root apices were supposed to be at fault, very little constitutional benefit followed either extraction or treatment.

Considered from a bacteriologic or biologic viewpoint, the writer is at this time not at all convinced that these areas, commonly called infected, merely because they show radio translucent areas, always are infectious or that organisms of harmful influence are at all times even present. Obviously these translucent areas may be produced by many agencies other than bacteria. The use of arsenical applications to a pulp, or the introduction of coagulating substances, commonly used in treatment, such as formaldehyde, easily might coagulate the lymph in the lymph spaces about these root ends and produce these radio translucent areas without meaning an infection at all; further, the method by which the harmful bacterial invasion would necessarily have to take place from these granulomas is one which the writer is not ready to generally admit at this time.

True, we hear of many spontaneous beneficial results from tooth extraction in many suspected cases; but the writer has seen also many instances in which teeth were removed without any constitutional benefit whatever following such procedure. If cases are presented where oral foci of infection actually are shown to be the cause of the secondary infection, or where all other sources of infection have been ruled out, and only as a matter of last resort these suspected areas are to be removed for the benefit of general health by extraction of the teeth suspected, then let it be understood that the mere removal of the teeth, followed by a slight, careless scarification with a curette is not at all sufficient to produce the desired results, but the operation must be concluded by vigorous surgical interference, such as properly cutting away any diseased bone and all granulomatous tissue that may have been suspected to be present through the X-ray examination. Many of these so-called granulomas, which have been the cause of removal of many teeth, can and have been treated with the result that later pictures showed these areas completely healed, or on the way to be completely healed.

The ruthless, unscientific extraction of teeth is entirely inadvisable, in the estimation of the writer. Much more beneficial clinical results have been obtained in the cleaning up of superficial infections, such as are found in unclean mouths, which are due to neglect or to pyorrhea, by simple prophylactic measures, than are found as the result of extraction after diagnosis of root infection. I am reminded of a case under observation in our Chicago laboratories at the present time, where a woman of about thirty-five years of age, suffering from arthritis, was advised to undergo prophylactic treatment; and a blood examination showed before treatment:

| | |
|---|---|
| Erythrocytes .................... | 500,000 |
| Leucocytes ...................... | 13,000 |
| Hemoglobin ..................... | 90% |
| Color Index ..................... | 80% |
| | |
| Polymorphonuclear .............. | 58% |
| Small Mononuclear .............. | 37% |
| Large Mononuclear .............. | 3% |
| Transitional ..................... | ...... |
| Eosinophiles. .................... | 2% |
| Basophiles. ...................... | ...... |

Red cells normal in shape and size. Stain well.

and after treatment:

| | |
|---|---|
| Erythrocytes .................... | 5,400,000 |
| Leucocytes ...................... | 730,000 |
| Hemoglobin ..................... | 1% |
| Color Index ..................... | 1% |
| Polymorphonuclear .............. | 68% |
| Small Mononuclear .............. | 26% |
| Large Mononuclear .............. | 4% |
| Transitional ..................... | 1% |
| Eosinophiles .................... | 1% |

This woman had her teeth X-rayed, and no less than eight teeth were found with radio-translucent areas, and, notwithstanding the fact that she only received local ordinary prophylactic treatment, and that nothing whatever, up to the present, at least, has been done to overcome any possible infection from the so-called granulomas, her improvement in the examination as far as blood differential was concerned, you will readily see was certainly striking; in fact, is now almost normal; and I fail to see that any further great improvement is to be expected following the treatment of these root apices as far as the blood picture is concerned. Her rheumatic condition has improved greatly and has almost disappeared. It should be mentioned, however, that she recently has undergone profuse eliminative treatment.

This is one of those cases where possibly the rheumatic condition may have been due to oral sepsis; not to any particular organism, but to a general albumin poisoning, for the organisms thus removed certainly were not of so infectious a nature as to locate as such in other surroundings and there set up their virulent activities. If they had been, the late removal of the original seat of infection hardly would have been sufficient to eradicate them in their new location nor benefit the blood picture. I believe that in such cases a low-grade poisoning of albumoses is established, and that activities of antibody production is so hindered as to allow deleterious bodily influences to work havoc without any direct infection spreading from the original source of disease. This toxemia being removed, allows the bodily functions to return to normal, and the disappearance of arthritic symptoms readily might be expected to follow.

A sequential disease should be traced to its proper source in a thoroughly scientific manner before pronouncing the final diagnosis.

It also should be determined as to which of the two infections was first present. A synchronous existence of two disturbances does in no way prove that one was dependent on the other; they may both or either one have been caused by a third bacterium or toxin, and therefore may be entirely independent occurrences.

We are all seeking the truth and that can only be obtained by true scientific study, entirely unbiased by prevailing opinions.

